# A Comparative Analysis of Visual Inspection With Acetic Acid, Cervical Cytology, and Histopathology in the Screening and Early Detection of Premalignant and Malignant Lesions of the Cervix

**DOI:** 10.7759/cureus.29762

**Published:** 2022-09-29

**Authors:** Japhia David, Vrunda Joshi, Devarajan Jebin Aaron, Priya Baghel

**Affiliations:** 1 Obstetrics and Gynaecology, Gajra Raja Medical College, Gwalior, IND; 2 Surgery, Jawaharlal Institute of Postgraduate Medical Education and Research, Pondicherry, IND

**Keywords:** visual inspection with acetic acid, pap smear, histopathology, cervical cancer, acetowhite

## Abstract

Introduction: The incidence of cervical cancer and related mortality is growing worldwide. The natural history of disease progression ranges from 10 to 20 years. Hence, effective screening can help in the early detection and prevention of fatal complications. This study aims to (1) compare the sociodemographic characteristics of the women with malignant and premalignant lesions of the cervix, (2) collate the accuracy of visual inspection of the cervix with acetic acid (VIA) with Pap smear cervical cytology in the early detection of premalignant and malignant lesions of the cervix, and (3) standardize it with histopathology, a gold standard screening tool.

Methods: This study was carried out in the Department of Obstetrics and Gynaecology, Gajra Raja Medical College, Gwalior, India, from October 2020 to March 2021 including all the sexually active women of the reproductive age group and postmenopausal women attending the gynecology outpatient department and the indoor admitted patients. A total of 500 patients were included in the study. The women were subjected to a Pap smear followed by VIA. Punch biopsy was taken from the acetowhite regions and sent for histopathological examination. The women with abnormal cervical cytology results also underwent a biopsy and histopathological examination.

Results: On comparative analysis, the sensitivity and specificity of Pap smear cytology were found to be 89.5% and 65.2%, respectively. The sensitivity and specificity of VIA were found to be 94.7% and 88%, respectively. The overall accuracy of VIA testing (93.2%) is more significant than that of Pap smear (68%).

Conclusion: According to our study, it is found that visual inspection with acetic acid is more diagnostically accurate than Pap smear cytology. Hence, VIA testing could be implemented as a primary screening tool with credence. Also, as learned from our study, the premalignant and malignant lesions are more common among elderly women living under a low socioeconomic status. Hence, these groups of women must be outreached and covered through effectively targeted screening programs.

## Introduction

The incidence of cancer and related mortality is growing worldwide. Cervical carcinoma is the 10th most common malignancy worldwide and is the ninth leading cause of cancer-related death in women according to the latest GLOBOCAN data 2020 [1]. According to the latest World Health Organization (WHO) data, there are 570,000 new cases of cervical cancer diagnosed worldwide and 311,000 cancer-related deaths [2].

Human papilloma Virus (HPV) infection is considered the most common cause of cervical carcinoma in developing nations. It is shown that types 16, 18, 31, 33, and 45 are most frequently associated with cervical carcinoma. Other risk factors like smoking, increased parity, lower socioeconomic status, and multiple sex partners have also been identified. The high prevalence of HPV infection in association with the lack of effective screening and treatment of this infection leads to the increased burden of cervical carcinoma [3].

The natural history of the progression of the disease ranges from 10 to 20 years. According to the study by Obrzut et al., the overall five-year survival rate of carcinoma cervix treated by hysterectomy is found to be 77.5% [4]. Hence, the diagnosis of the disease earlier can prevent fatal complications. The premalignant changes in the cervix can be detected by methods like Pap smear cytology and liquid-based cytology (LBC) along with HPV DNA testing [5]. Visualization of the cervix is also performed as one of the screening procedures where the cervix is examined for the acetowhite regions after the application of 3%-5% acetic acid with a further evaluation with the application of Lugol’s iodine. This can be followed by colposcopic examination and directed biopsies. However, histopathology is considered the gold standard in the diagnosis of premalignant and malignant lesions of the cervix [6]. This study aims to compare the sociodemographic characteristics of the women with malignant and premalignant lesions of the cervix, collate the accuracy of visual inspection of the cervix with acetic acid (VIA) with Pap smear cervical cytology in the early detection of premalignant and malignant lesions of the cervix, and standardize it with histopathology, a gold standard screening tool.

## Materials and methods

This prospective cross-sectional study was carried out in the Department of Obstetrics and Gynaecology, Gajra Raja Medical College, Gwalior, Madhya Pradesh, India, from October 2020 to March 2021. The patients including all the sexually active women of the reproductive age group and postmenopausal women with any symptoms attending the gynecology outpatient department and the indoor admitted patients were included in the study. Written informed consent was taken from all the participants of the study. Women who refused to give consent, known cases of carcinoma cervix, and women with acute severe bleeding in whom Pap smear cytology or VIA is impossible were excluded from the study. A total of 500 patients were included in the study.

Under well-informed consent, after proper history taking, the women were subjected to examination. Pap smear cytology was preferred as the first test in terms of its cost-effectiveness and easy availability from our institution compared to other cytological tests. Under all aseptic precautions, a Cusco's bivalve speculum was introduced, and the cervix was visualized. Any excessive vaginal discharge was gently removed, and initially, a Pap smear was taken from the squamocolumnar junction using Ayre’s spatula and a smear was prepared. A conventional Pap smear technique was used. The Pap smear slide was initially fixed by dipping it in 95% ethanol solution for about 15 minutes. After rehydration using tap water, the staining procedure is done using the polychromic Papanicolaou stain. The polychromic Pap stain contains the major reagents under three preparations, which include the Harris hematoxylin, Orange Green-6 (OG-6), and the Eosin Azure (EA-50) dyes. The slide is placed initially in the hematoxylin dye for a minute followed by a tap water rinse. This preparation is given a 10 times dip in 95% ethanol and the placement of slides in OG-6 dye for 1.5 minutes afterward. This is followed by a 10 times dip in 95% ethanol and subsequent placement of the slides in EA-50 dye for 1.5 minutes. This is again followed by a 10 times dip in 95% ethanol for two turns and subsequent placement of the slides in 100% ethanol for a minute. This is finally replaced with a transparent xylene preparation making it suitable for the final mounting. These Pap smears were analyzed and reported according to the Bethesda system of classification.

All the cases undergoing Pap smear testing consequently underwent visual inspection with 5% acetic acid (VIA). Sharp, well-described acetowhite regions touching the squamocolumnar junction were considered positive. Punch biopsy was taken from the acetowhite regions and sent for histopathological examination (HPE). The reports of cervical cytology were collected, and those who had abnormal results underwent biopsy and histopathological examination also to aid the comparative analysis with VIA. The data collected were analyzed using SPSS software, version 20.0, manufactured by the SPSS, an IBM company, which is headquartered in Chicago and integrated with Delaware, United States. A comparison between Pap smear cytology, VIA, and histopathology was done. The sensitivity, specificity, positive predictive value, and negative predictive value were calculated to see the diagnostic accuracy of the screening tests. The power of the study was calculated to be 0.84, and thus, the percentage of power was found to be 80%.

This study has been approved by the Institutional Ethics Committee (IEC), Gajra Raja Medical College, Gwalior, Madhya Pradesh, India, with the approval number "59/IEC-GRMC/2018."

## Results

Distribution of cases based on sociodemographic characteristics

On analysis of the cases based on age, the average age of the sampled population was found to be 46.54 years. The maximum value was 95 years of age, and the minimum value was 14 years of age. Accordingly, the range was calculated to be 81 years. The maximum number of the diagnosed premalignant lesions (61.5%) and malignant lesions (27.2%) belonged to the age group > 55 years, and the minimum number of cases was found in the age group less than 20 years.

On analyzing the distribution of cases based on socioeconomic status, the maximum number of the diagnosed women belonged to the lower socioeconomic groups, the upper lower class and lower middle class, with premalignant (46.8%) and malignant lesions (13.9%).

On analysis of the cases based on the age at marriage and intercourse, it was found that the majority of the diagnosed cases of both groups of premalignant (54.9%) and malignant lesions (16.9%) had their age of marriage at <21 years, indicating an earlier age of sexual intercourse.

On analysis of the cases based on parity, both groups of the premalignant (66.7%) and malignant lesions (48.1%) had a parity of >5. All these findings were calculated to be statistically significant with a p-value of <0.05 with an exception of the categorization of the premalignant lesions based on socioeconomic status, which showed no significance (Table 1).

**Table 1:** Distribution of cases based on sociodemographic characteristics

Patient characteristics	Number of patients (n = 500)	Premalignant lesions (n = 205)		Malignant lesions (n = 57)		Chi-square value	p-value
		Number	Percentage	Number	Percentage		
Age (Years)							
<25	56	7	12.5	0	0	Premalignant: 62.79; malignant: 64.452	Premalignant: <0.05; malignant: <0.05
26-35	93	26	28.0	2	2.2		
36-45	115	34	29.6	1	0.9		
45-55	100	54	54.0	17	17.0		
>55	136	84	61.8	37	27.2		
Socioeconomic status (Modified Kuppuswamy socioeconomic scale)							
Upper	10	2	20.0	0	0	Premalignant: 5.55; malignant: 9.96	Premalignant: >0.05; malignant: <0.05
Upper middle	52	18	34.6	0	0		
Lower middle	201	79	39.3	28	13.9		
Upper lower	175	82	46.8	23	13.1		
Lower	62	24	38.7	6	9.7		
Age at marriage and intercourse							
<21 years	213	117	54.9	36	16.9	Premalignant: 29.765; malignant: 11.119	Premalignant: <0.05; malignant: <0.05
>21 years	287	88	30.7	21	7.3		
Parity							
<1	112	51	45.5	16	14.3	Premalignant: 9.957; malignant: 41.768	Premalignant: <0.05; malignant: <0.05
1-5	361	136	37.7	28	7.8		
>5	27	18	66.7	13	48.1		

Distribution of cases according to presenting complaints

**Figure 1:**
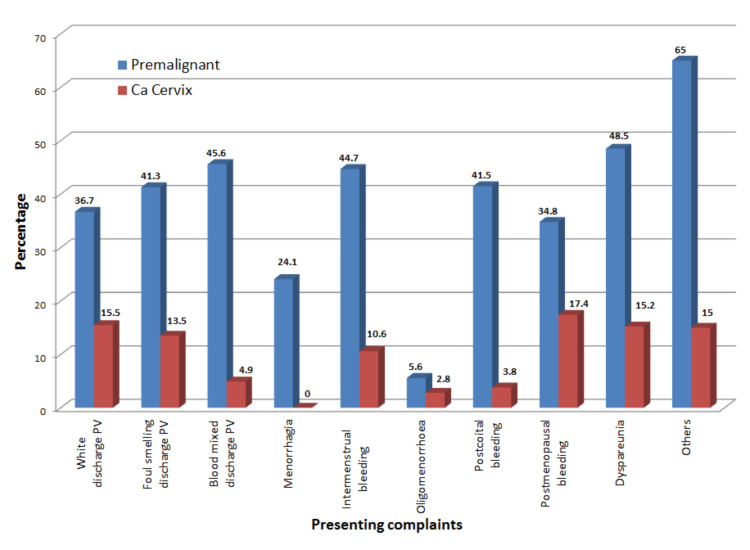
Distribution of cases according to presenting complaints

In our study, the majority of the diagnosed premalignant lesions (65%) had presented with vague symptoms of abdominal pain, loss of appetite, and weight. Abnormal bleeding-related complaints like blood mixed discharge per vaginum (45.6%), intermenstrual (44.7%), and postcoital bleeding (41.5%) were present in an appreciable number of individuals diagnosed with premalignant lesions. The majority of the diagnosed malignant lesions had presented with postmenopausal bleeding (17.4%), which is shown in Figure 1.

Distribution of cases according to Pap's smears’ results

**Figure 2:**
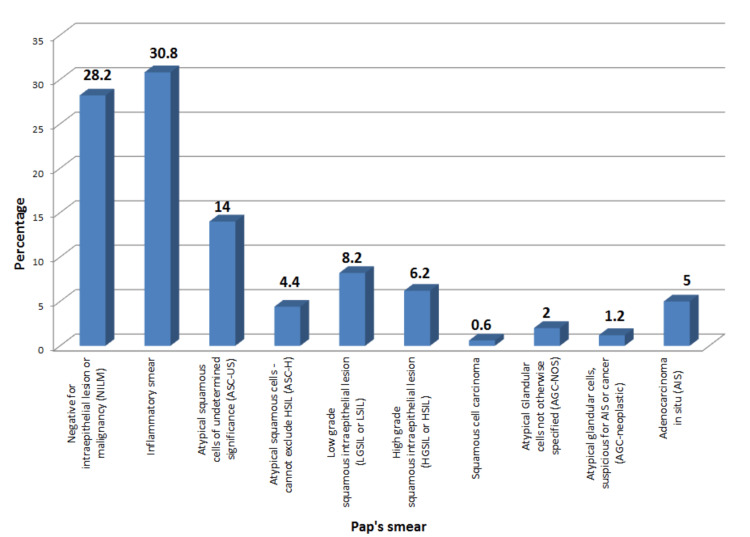
Distribution of cases according to Pap's smears’ results

In our study, among the 500 patients who had undergone Pap smear cytology, the majority were negative for intraepithelial lesions or malignancies (NILM, 28.2%). About 41% of cases had reports suggestive of a premalignant or malignant lesion, of which the majority of cases had atypical squamous cells of undetermined significance (ASC-US, 14%). These 205 patients were then subjected to histopathological examination (Figure 2).

Distribution of cases according to the result of the VIA

**Figure 3:**
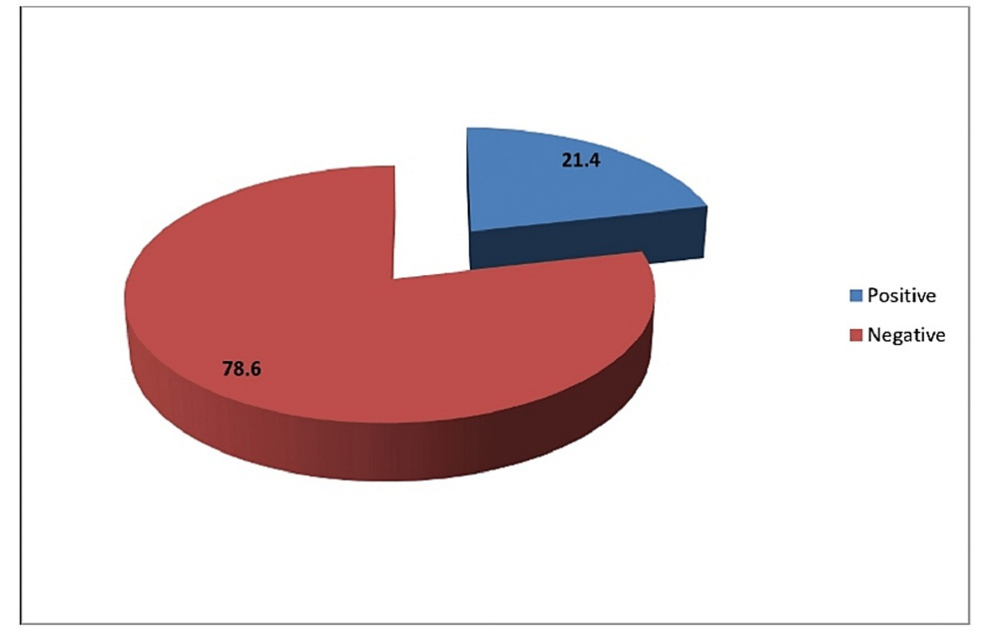
Distribution of cases according to the result of the VIA VIA: Visual inspection of the cervix with acetic acid.

In our study, among the 500 patients who had undergone the VIA test, the majority were negative (78.6%) for the test, and the rest (21.4%) presented with a positive test. These patients were subjected to histopathological examination (Figure 3).

Distribution of cases according to HPE reports

**Figure 4:**
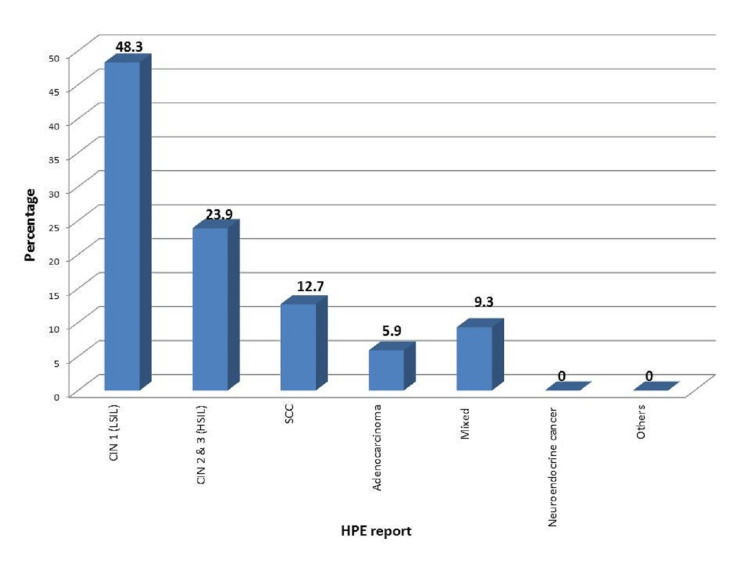
Distribution of cases according to HPE reports HPE: Histopathological examination; LSIL: Low-grade squamous intraepithelial lesion; HSLI: High-grade squamous intraepithelial lesion; SCC: Squamous cell carcinoma.

It was found in our study that all the women who had positive VIA also had abnormal Pap smear results. Hence, the net number of cases who underwent a biopsy was 205 including the women with positive VIA and abnormal Pap smear. On analysis, it was found that the majority were diagnosed with premalignant lesions, CIN 1 (low-grade squamous intraepithelial lesion [LSIL], 30.7%) followed by CIN 2 and 3 (high-grade squamous intraepithelial lesion [HSIL], 23.9%). About 27.8% of cases were diagnosed with malignant lesions. Among those diagnosed with carcinoma, the majority of the cases were squamous cell carcinoma (12.7%), which is shown in Figure 4.

Comparison of Pap's smear cytology with histopathology

**Figure 5:**
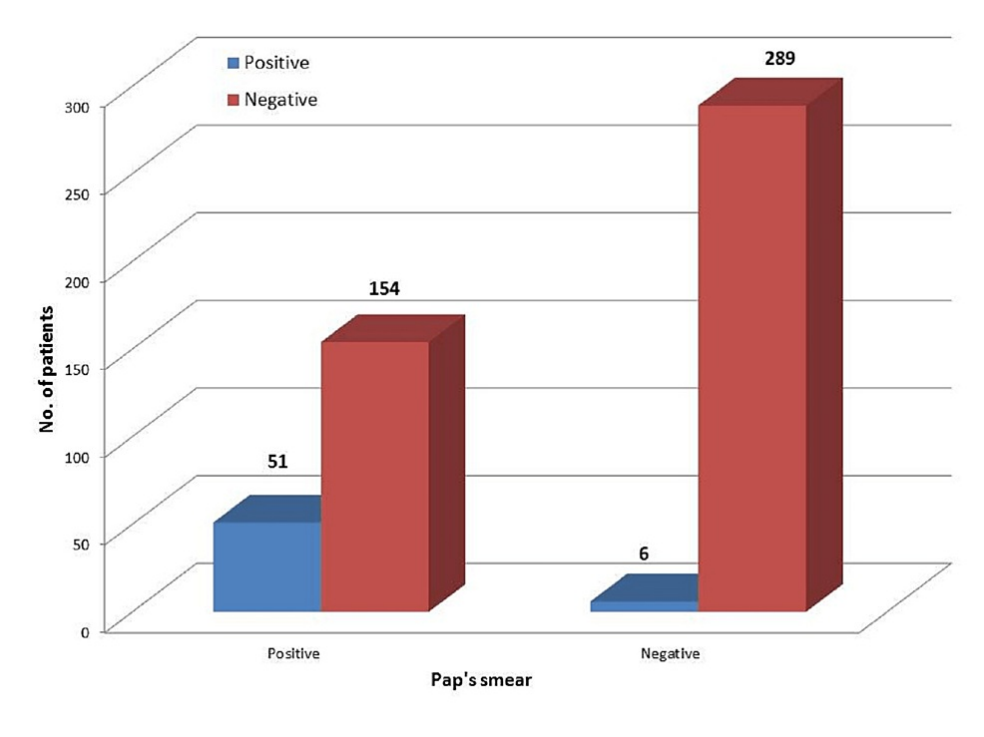
Comparison of Pap's smear cytology with histopathology

In the comparison of Pap smear cytology results with histopathology, the sensitivity was found to be 89.5%, and the specificity was found to be 65.2%. The positive predictive value of Pap smear cytology was found to be 24.9%, and the negative predictive value of the test is 65.2%. The overall accuracy of Pap smear cytology was 68% (Figure 5).

Comparison of VIA with histopathology

**Figure 6:**
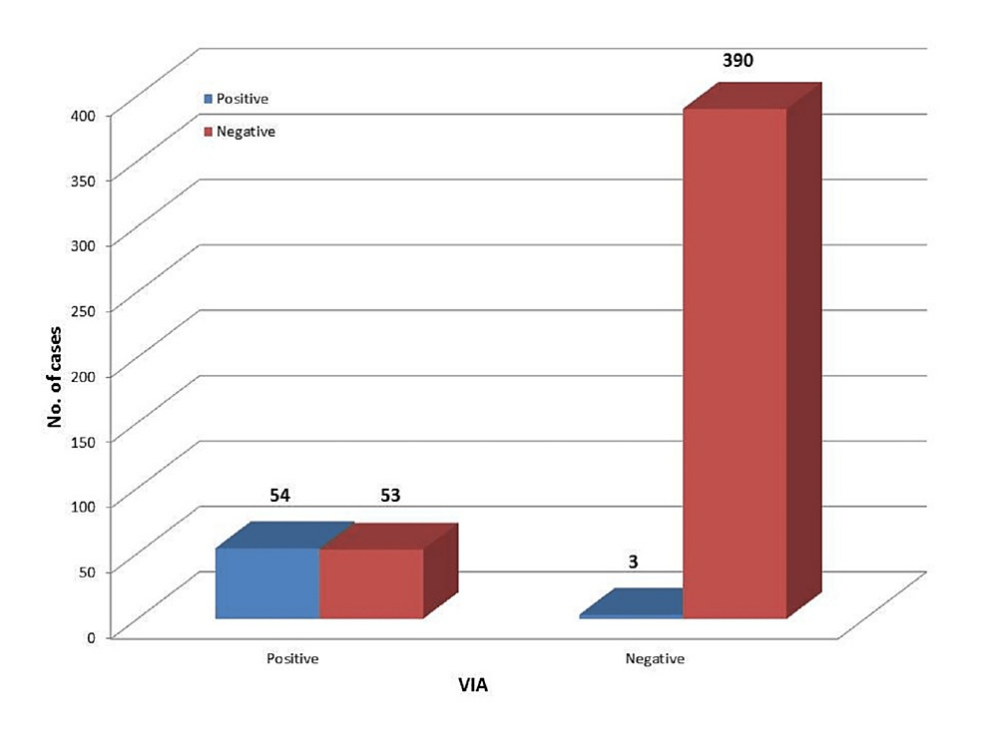
Comparison of VIA with histopathology VIA: Visual inspection of the cervix with acetic acid.

In the comparison of VIA results with histopathology, the sensitivity was found to be 94.7%, and the specificity was found to be 88%. The positive predictive value of VIA was found to be 73.2%, and the negative predictive value of the test is 95%. The overall accuracy of visual inspection with the acetic acid test is 93.2% (Figure 6).

## Discussion

This prospective, comparative analysis of Pap smear cytology and VIA considering histopathology as the gold standard test identifies VIA as a feasible investigation to identify the premalignant and malignant lesions of the cervix.

In our study, the study population included all the women presenting with gynecologic-specific symptoms. On analysis, the majority of the women of both the category with premalignant (61.8%) and malignant lesions (27.2%) belonged to the >55-year age group. Most of the women diagnosed with premalignant lesions and malignant lesions belonged to the lower socioeconomic groups, the upper lower (46.8%) and the lower middle (13.9%) classes, respectively. In comparison with a similar study by Bhattacharyya et al., the majority of the women with premalignant lesions belonged to the age group of 40-49 years, and the malignant lesions belonged to the age group of 50-59 years. The majority of the positive premalignant cases belonged to the low socioeconomic group. These findings are more or less comparable to our study [7]. However, a similar study by Egede et al. suggests that there is not much statistical significance in socioeconomic status [8]. These patterns of distribution might vary among different places due to the different geographic allocations and ethnic differences.

On the analysis of cases according to the age of marriage, the majority of women with premalignant (54.9%) and malignant lesions (16.9%) belonged to the <21-year age group, which is comparable to the study by Bhattacharyya et al., where the majority of the cases belonged to <18 years of age. It is also reported that the incidence of CIN increases with the increased duration of marital life, indicating an earlier age of marriage and earlier sexual intercourse. The highest number of CIN (30%) and cervical cancer (6%) are reported in the group with marital life of more than 20 years.

According to the distribution of cases based on parity, our study reports an increased incidence of premalignant (66.7%) and malignant lesions (48.1%) in cases with increased parity of >5. This is in similarity to the study conducted by Bhattacharyya et al., where it is reported that there is an increased incidence of CIN (54%) in women with parity >2 [7]. A population-based study by Hinkula et al. also suggests that the incidence of CIN and cervical carcinoma was greater in grand multiparous women [9]. The reason attributed to the higher incidence is that the transformation zone is maintained in the ectocervix in multiparous women. During pregnancy, there is an increased multiplication of squamous metaplastic cells, and these immature squamous metaplastic cells are more susceptible to HPV infection and other carcinogenic agents, leading to the pathogenic process of cervical carcinoma as studied by Muñoz et al. [10].

The majority of the women in our study with premalignant lesions had presented with vague symptoms of abdominal pain, loss of appetite, and weight (65%). Abnormal bleeding-related complaints like blood mixed discharge per vaginum (45.6%), intermenstrual (44.7%), and postcoital bleeding (41.5) were present in an appreciable number of individuals diagnosed with premalignant lesions. The majority of the diagnosed malignant lesions had presented with postmenopausal bleeding (17.4%). This is comparable with the six-year study by Eze et al. conducted to analyze the presenting symptoms of cervical cancer where the majority of the women presented with abnormal menstrual bleeding (86.9%) [11]. Similarly, in a cross-sectional survey analysis by Mwaka et al., it was identified that the majority had reported intermenstrual bleeding (85%) and postmenopausal bleeding (84%) as the significant symptoms [12]. Hence, women presenting symptoms suggestive of abnormal forms of bleeding should be given priority. Also, as learned from our study, vague symptoms may be associated with premalignant cervical lesions. Hence, all women must be subjected to vigilant cervical screening.

**Figure 7:**
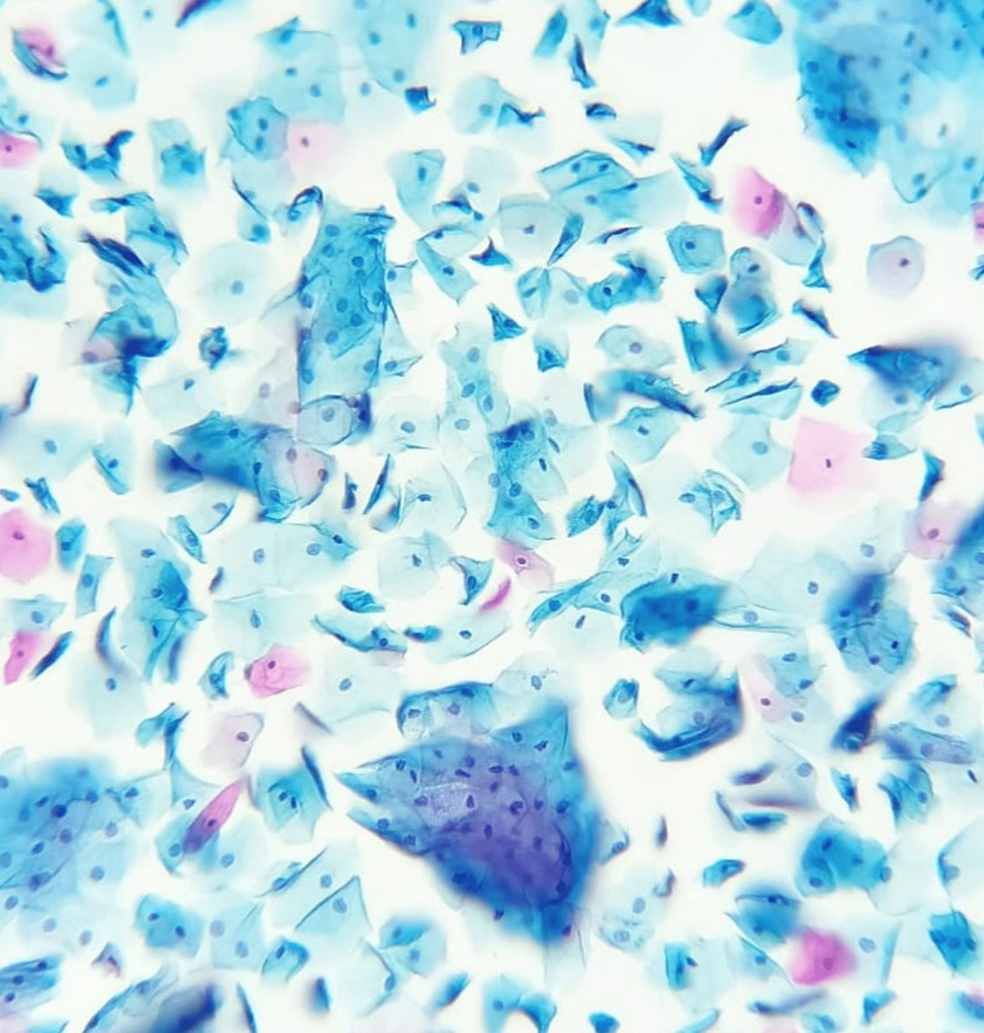
Cytologic picture of cervical Pap smear showing negative for intraepithelial lesion or malignancy

**Figure 8:**
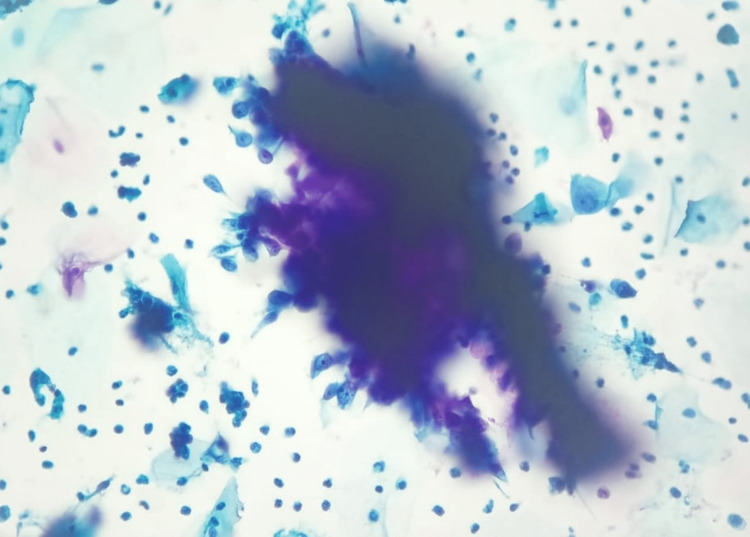
Cytologic picture of cervical Pap smear showing endometrial atypical glandular cells

On the analysis of the cases according to the Pap smear results, 41% of the women had abnormal Pap smear results; 14% of the abnormal results showed atypical squamous cells of undetermined significance (ASC-US). This is comparable to the study conducted by Vahedpoor et al., which reports an ASC-US incidence of 17% among the 21% of abnormal Pap smear results [13]. In a large study conducted by Sachan et al. on 1650 women, it was found that 48.8% were NILM and 42.6% had inflammatory smear, whereas in our study, 28.2% had presented with NILM and 25.6% had presented with an inflammatory smear. The highest incidence reported was of LSIL, which was found to be 5.09% followed by ASC-US (2.9%). This is a little contrary to our study. Some of the reasons attributed to the increased incidence of cervical cytological abnormalities are cultural differences, age of the individuals, and related infections [5]. The cytologic images of NILM and a quite noticeable positive case of endometrial atypical glandular cells are presented for reference (Figures 7, 8).

In our study, on the analysis of VIA testing of 500 cases, it was found that 21.4% tested positive for the test. This, in comparison to a similar study by Poli et al., where a single VIA testing was done on 18,869 women over seven years resulted in 2030 women testing positive for VIA. This was equivalent to 10.75%, which is a little lower than our study [14]. Our study population had more incidence of premalignant and malignant lesions. However, in comparison with an Indian study by Sankaranarayanan et al. of 4444 screened women, it was found that 24.2% of women were low‐threshold VIA positive and 15.8% of high‐threshold VIA positive, which is equivalent to the results of our study [15]. Indian population has more incidence of carcinoma cervix.

The WHO-approved feasible strategy in cervical cancer screening and prevention is the “screen and treat” approach, where the women who tested positive for VIA are given cryotherapy treatment at the same visit. This cost-effective feasible approach can significantly reduce the number of further follow-ups and the loss of follow-up status [16].

**Figure 9:**
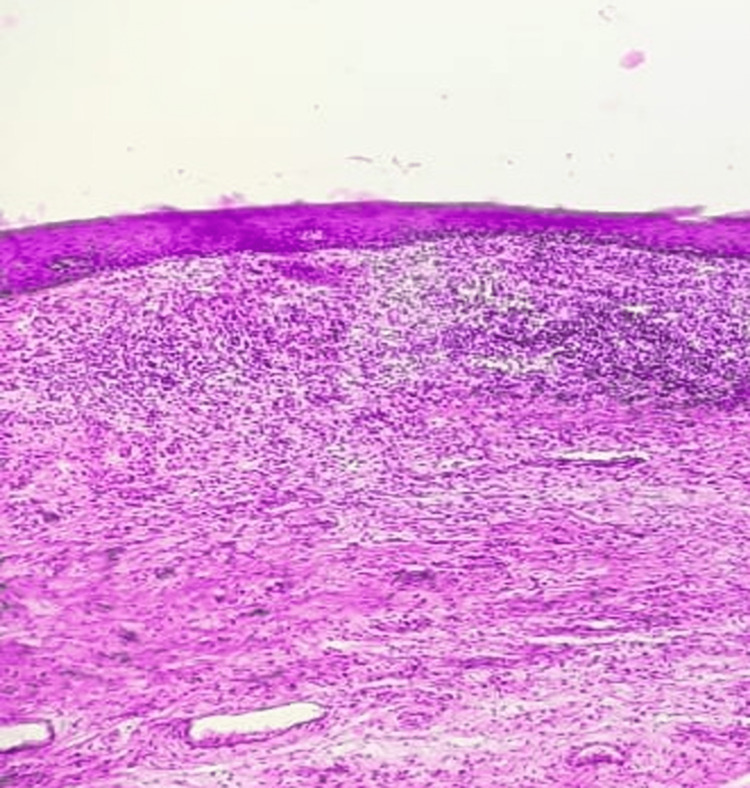
Histopathologic picture of a normal cervix showing stratified squamous epithelium

**Figure 10:**
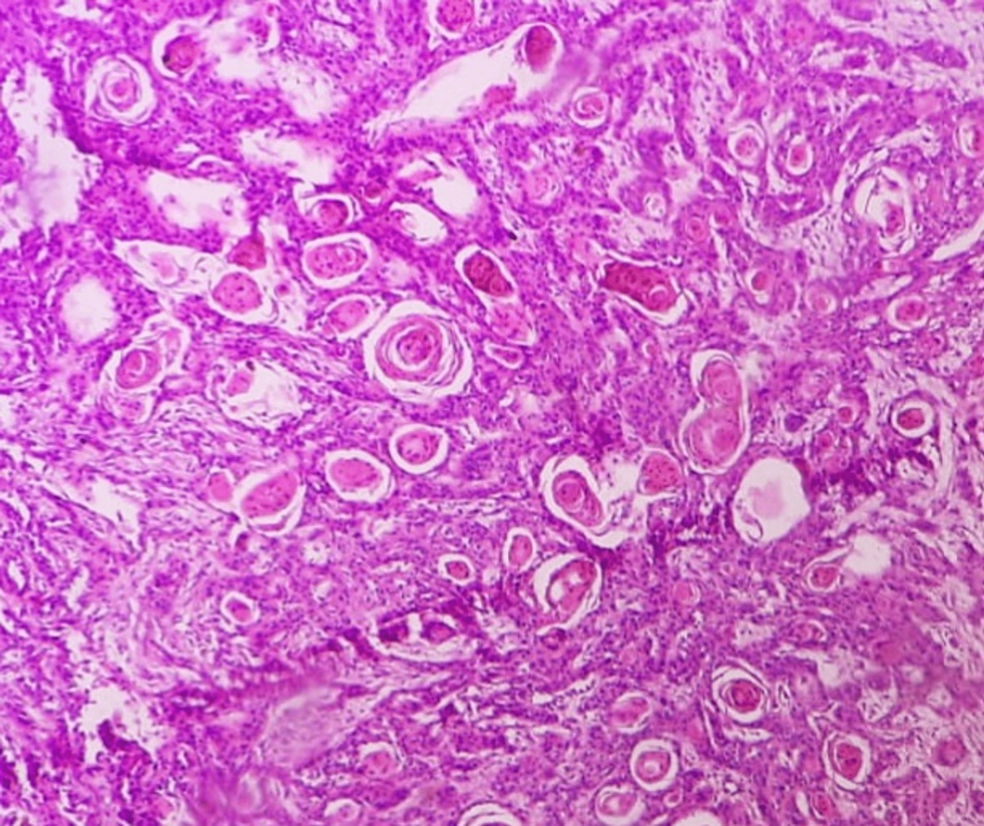
Histopathologic picture showing a well-differentiated keratinizing squamous cell carcinoma

**Figure 11:**
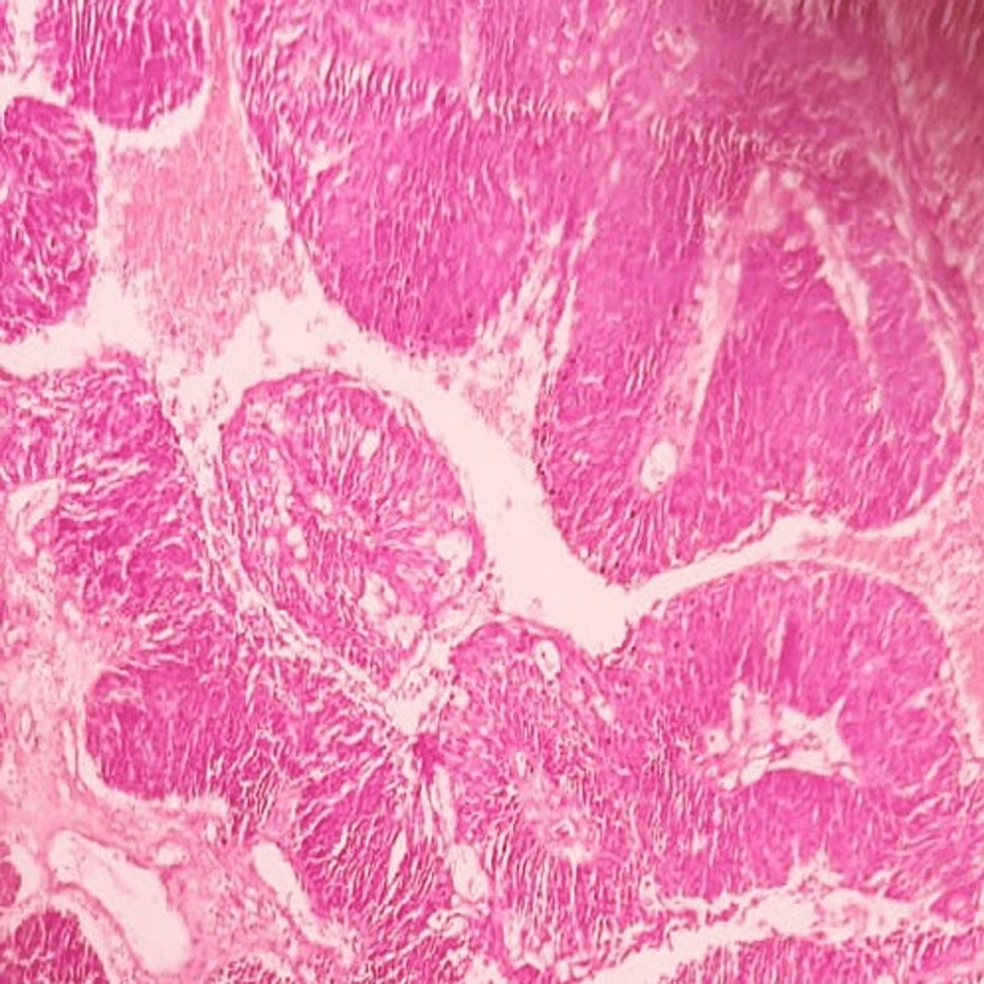
Histopathologic picture showing non-keratinizing squamous cell carcinoma

For over 50 years, biopsy and histopathological examination is considered the gold standard in diagnosing cervical cancer [17]. Owing to it, considering biopsy and histopathological examination the gold standard, 57 cases (11.4%) in our study were diagnosed with carcinoma cervix. Among these, the majority of the cases were diagnosed with squamous cell carcinoma (12.7%) followed by mixed carcinoma (9.3%). The histopathologic images of a normal cervix and positive cases of squamous cell carcinoma are presented for reference (Figures 9-11).

On comparing Pap smear cytology results with histopathology, the sensitivity of our study is found to be 89.5%, and the specificity was found to be 65.2%. The positive predictive value of the Pap smear was found to be 24.9%, and the negative predictive value of the test was 65.2%. The overall accuracy of Pap smear cytology is 68%. However, in comparison to VIA results with histopathology, the sensitivity of VIA in our study was found to be 94.7%, and the specificity was found to be 88%. The positive predictive value of Pap smear cytology was found to be 73.2%, and the negative predictive value of the test was 95%. The overall accuracy of visual inspection with the acetic acid test is 93.2% indicating that VIA is superior to Pap smear cytology. In a similar study by Bhattacharyya et al., the sensitivity and specificity of Pap smear were found to be 52% and 95%, respectively. The positive and negative predictive values of Pap smear were found to be 45% and 96%, respectively. The sensitivity and specificity of VIA were found to be 89% and 87%, respectively. The positive and negative predictive values of VIA were found to be 32% and 99%, respectively. This study concludes that VIA is more sensitive than Pap smear, which is comparable to our study. But it was derived that VIA is less specific than Pap smear, which is contrary to our study. The overall accuracy of Pap smear was 93% and VIA was 87%, which is contrary to our study. The reason attributed to low specificity is the increased incidence of acetowhite areas in inflammation and immature metaplasia [7].

In a similar study by Sankaranarayan et al., the sensitivity and specificity of Pap smear were found to be 81.9% and 87.8%, respectively. The positive and negative predictive values of Pap smear were found to be 19.1% and 99.3%, respectively. The sensitivity and specificity of VIA were found to be 82.6% and 86.5%, respectively. The positive and negative predictive values of VIA were found to be 17.5% and 99.3%, respectively. This study concludes that VIA is more sensitive than Pap smear, which is comparable to our study. But VIA is considered a little less specific than Pap smear, which is contrary to our study. The reason attributed is the need for training and standard criteria for VIA test positivity to improve the test quality of VIA. The inability to detect endocervical disease is considered a likely limitation of VIA [15].

Compared to a similar study by Egede et al., Pap smear was found highly sensitive (80%) compared to VIA (73.3%) contrary to our study. But VIA was found highly specific (96.5%) than Pap smear (91.8%), and the overall accuracy of VIA (93%) was also found to be higher than Pap smear (90%), similar to the results of our study. It also suggests that the overall accuracy increases to 99% when Pap smear and VIA are done as combined testing [8].

In a similar study conducted by Vahedpoor et al., the sensitivity of VIA (94.6%) was higher compared to Pap smear (29.7%), which is similar to our study. But Pap smear (85.5%) was found more specific than VIA (81.6%). The reason for the low specificity is that acetowhite lesions due to cervical polyps and metaplasia were considered positive leading to higher false positive results. Hence, these lesions should be ruled out first, and knowledge regarding these lesions should be improved to improve the specificity [13]. It was also found that the sensitivity in the detection of high-grade lesions can be improved up to 100% by combined usage of Pap smear and VIA [17].

Furthermore, it could be noted that the usage of Pap smear cytological testing, however, has added benefits like detection of sexually transmitted infections as described by Jayapalan et al. in their study. The commonly detected infections include HPV infection and trichomoniasis. Koilocytosis in an asymptomatic woman, an abnormality detected by Pap smear cytology often points toward HPV infection. Hence, the women at risk for cervical carcinoma due to the initial agent HPV can be picked up early and treated according to these findings. Also, nuclear abnormalities like melting or rupture of nuclear membrane along with other inflammatory cellular features could point toward endocervicitis. The causative agents for this endocervicitis could be species of *Chlamydia*, *Neisseria*, *Trichomonas*, *Mycoplasma*, etc. Hence, these infections could also be picked up through a Pap smear screening [18].

## Conclusions

Carcinoma cervix has a long natural history of progression to the malignant stage. This makes the disease amenable to early detection by screening and prevention of the disease course. According to our study, it is found that VIA is more diagnostically accurate than Pap smear cytology. Hence, VIA testing could be implemented as a primary screening tool with credence. Therefore, all health care providers must be trained systematically in performing the VIA procedure efficiently. Also, as learned from our study, premalignant and malignant lesions are more common among elderly women living under a low socioeconomic status. Hence, these groups of women must be outreached and covered through effectively targeted screening programs. These small measures could alleviate the burden of carcinoma cervix.
